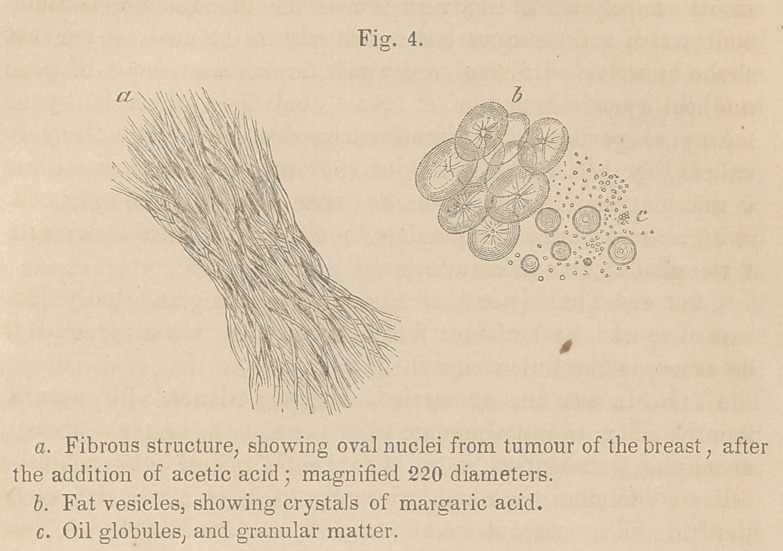# Microscopical Observations of Tumors

**Published:** 1851-12

**Authors:** John H. Brinton

**Affiliations:** Philadelphia


### MEDICAL EXAMINER,

AND

### RECORD OF MEDICAL SCIENCE.

NEW SERIES.—NO. LXXXIV.—DECEMBER, 1851.

---

### ORIGINAL COMMUNICATIONS.

---

### *Microscopical Observations of Tumors.* By JOHN H. BRINTON, of Philadelphia.

Cancer is to be met with in almost every portion of the human frame, we find it involving alike skin, mucous membrane, muscles, fibrous tissue, and nerve, and even deposited in the medullary cavities of the bones. If then the question be put, what are the elements of a cancerous tumor, the answer must be a general one: the cells of cancer infiltrated into the minute structure of any tissue. In examining therefore any suspected growth, it becomes necessary for us to bear constantly in mind the appearances presented by the part, not only in a state of health, but also when affected by any other disease, which may yet be foreign to cancer. Let us glance for a moment at the various kinds of cells which may meet the view of the microscopist in his pathological examinations, and which, unless the character of each be carefully studied, may easily be confounded with those of cancer. We have the epithelial cell, the fusiform and the cartilage cell, the compound granular cell as described by Bennett, fat vesicles, glandular cells, and the pus cell. The difference between these various cells, and those of cancer, will be described as they present themselves in each case. We have already seen in case number two, (in the preceding number of this Journal,) the fusiform corpuscle as met with, when going to the formation of fibres, and in the same case we have also represented the fat vesicles as they ordinarily appear.

How common is it to hear such remarks as the following from those who are called, to use a common phrase, "unbelievers in the microscope." "You say this is cancer; what is your standard of cancer, whence is it derived, and what are your data? Have you any? or has it been arbitrarily assumed, that this is cancer, and that is not?" Now these are natural questions, and should be answered. The only true and practical standard of cancer, is that deduced from the careful and continued examination of such cases whose malignancy is undoubted; those cases which destroy life, either directly, or by returning after repeated extirpation. Now it will be found, that all those who have made this subject practically their study, will, as the result of their own investigations, establish for themselves a standard of their own; and moreover, if the results of all experienced modern observers be compared together, it will be evident that although they may differ in minutiæ, they still agree on the main, important, and practical points. Cancerous tumors may be arranged for the convenience of study into three great classes; first, the scirrhus or hard cancer; second, the encephaloid or soft cancer; and third, the colloid or gelatiniform cancer; the first two forms seeming to differ from each other merely in the presence of a greater or less proportion of fibrous tissue; many fibres and few cells constituting scirrhus; few fibres and many cells forming encephaloid. Scirrhus can not be distinguished from fibrous tumors by the unassisted eye; in reality scirrhus is only a fibrous tumor, with one element superadded, and that element is the cells of cancer; the arrangement of these cells is peculiar, they may either be infiltrated between the fibres, or else be gathered together in cysts, as will be seen in the case about to be described.

In encephaloid, the cells are in great abundance; and in consequence, the proportionate absence of fibrous tissue, renders the whole tumor much softer, approaching in truth as its name imports, to a brain-like consistency.

Schirrus and encephaloid may exist together, or rather scirrhus has a natural tendency to pass into encephaloid, by the more rapid developement of cells; so that we often find both of these forms of cancer united in one and the same tumor.

The synonymes of scirrhus are carcinomatous sarcoma, scirrhoma, fibrous cancer, and stone cancer; those of encephaloid, are medullary sarcoma, fungoid sarcoma, cerebriform cancer, soft cancer, spongy carcinoma, and many others too numerous to mention. While colloid cancer is described by writers as gelatiniform cancer, and areolar or gum cancer.

Of the whole class of cancers there are none that set more completely at defiance the skill of the surgeon, than those which are located in the uterus and ovaries; it is true that some years ago we had numerous French statistics of operations, which lessened much the mortality attending cases of carcinoma uteri; but further investigation sufficed to show that some error must have existed in the preparation of such statistics; and now, universally, cancers of the uterus are still considered as indomitable as before.

I have therefore selected the following case of carcinoma uteri, as one every way fitted to be the representative of a great class; it occurred in the practice of Dr. Bibighaus, of this city; who kindly furnished me with the specimen, and the following particulars.

Mrs. P., æt. 56, a widow, and the mother of several children; has been ill for several years, complaining of vague abdominal pains, feelings of lassitude, and all the other symptoms characteristic of uterine disease. During the year previous to her death, which took place in March last, the epigastric pains increased, becoming of a lancinating character; general derangement of the primæ viæ took place; the stomach sympathized to a marked degree with the uterus; constant vomiting occurred, and the patient finally sank. During all this period discharges of blood similar to the catameniæ occurred, although at irregular intervals. Upon post mortem examination, the thoracic organs were found healthy, as also were the intestines, liver, and kidneys, of both sides. The ovaries were enlarged, the fallopian tube of the right side had ulcerated off from the uterus, while a considerable deposit had taken place in that of the left side; the fundus of the uterus was much enlarged, and on its posterior portion softened, and infiltrated with a thick viscid fluid, of a dirty brown color, which could easily be squeezed out, by making pressure between the fingers. The os uteri was open and distended, its margins ragged and ulcerated; the cavity of the uterus was also enlarged, and was filled with a thick, greenish, fluid, fetid pus. The upper portion of the vagina was much ulcerated.

Fig. 3.

*a.* Cancer cells from uterus magnified 200 diameters.

*b.* Parent cell.

*c.* Cyst in fibrous tissue, filled with cells.

*d.* Pus corpuscles.

*e.* Action of Acetic acid on same.

Upon squeezing out from the cut fundus uteri, a portion of the viscid fluid it contained, and submitting it to the microscope, I found it to present the appearance shown at figure 3; numerous cells were to be observed, varying greatly in their shape and size, some being round, some oval, and others broad and flat, with a swallow-tailed extremity; these cells were larger than those occurring in most cases of cancer, except in those of rapid developement, or in an advanced stage; they measure from the 1-900th to the 1-2000th part of an inch in diameter, and were all possessed of an oval nucleus about the 1-3500th part of an inch in length, the nucleus varied however in proportion to the size of the cell; a number of the cells possessed in addition a nucleolus, appearing merely as a granule in the interior of the nucleus. These cells all floated as it were in a sea of granular matter. I observed also several parent cells, the mother cells of the German writers. (See *b*, fig. 3.) Two, three, four, and even more true cancer cells may often be distinguished in the interior of a parent cell. These mother cells are never met with except in cancers in an advanced state, or of rapid growth; by some they are supposed to split, so liberating the enclosed cancer cells. This, however, is merely an hypothesis, and has not yet been clearly proved. Many of the books lay down as a rule, that the parent cells which enclose in their interior the young, or daughter cells, are characteristic of encephaloid. Concerning this, Vögel, one of the earlier writers on this subject says, "that while he has not often seen these cells in other forms of cancer, still he cannot affirm that they always occur even in encephaloid." Upon examining the more solid parts of the fundus uteri, cysts filled with cancer cells were to be seen, occurring in the fibrous stroma of the uterus. (*c*.) These cysts were about the 1-500th part of an inch in diameter, and of about the same depth, and were formed by the interlacement of the fibres of the ordinary white fibrous tissue. The cells filling these cysts, could, when carefully examined, be seen to be possessed of nuclei. Compared with the other cells, they were however of small size, being only about the 1-2500th part of an inch in diameter.

At fig. 3, (*d*) I have represented the appearance of the pus cells, as met with in the cavity of the uterus. Pus is composed of two portions—pus corpuscles, or cells, and a liquor puris, resembling much the serum of the blood, in which the cells swim. The pus corpuscle varies in size, from the 1-2000th to the 1-3000th part of an inch in diameter, its medium size may be said to be about the 1-2500th part of an inch in diameter, that is about double the size of the corpuscle of the human blood; it is spherical, with a well defined and finely marked edge, and presents internally a granular appearance; by the addition of water the corpuscle increases in size, the water entering by endosmosis. Concentrated acetic acid dissolves instantly the cell wall, and brings into view a nucleus which is not visible in the corpuscle, unless under chemical action; this nucleus consists of two, three or four granules apparently connected together, (*e*, fig. 3.) How, then, do we distinguish the pus corpuscle from the cancer cell? The first is always globular, granular, and, unless acted upon chemically, presents no nucleus, and this nucleus, when so obtained, is peculiar to pus, and characteristic; the cell of cancer is generally much larger than the pus cell, is not often round, varies much in its shape, and presents an oval nucleus. Pus is sometimes confounded with mucous, but it has always seemed to me that the corpuscles of mucus are much larger than those of pus; and the chemical action of acetic acid upon them is by no means so perfect; the mucous corpuscles have a tendency to collect together, forming conglomerate masses, and their outline is granular and less finely marked than is that of the pus cell. With ordinary care it is possible to distinguish them apart with a tolerable degree of certainty.

Case 4.—The history of this case, which was kindly furnished to me by Dr. Jno. Wilson Moore, in whose practice it occurred, is the following:

E. G. M., æt. 48, unmarried, had experienced for several months prior to last June, occasional pains in the right breast; about the commencement of that month, a hard lump could be felt on examination, which gradually enlarged until the early part of July, when it was removed by Dr. Pancoast. The wound healed kindly, and the patient is now entirely recovered.

The breast, when removed, was of a medium size, and seemed to consist of a hard fibrous tumor, enveloped in a thick layer of fat, presenting to the view internally numerous dense nodulated bodies, of an almost cartilaginous consistency, crunching under the knife when cut; several cysts were also seen filled with a thick, oily fluid, varying in color from a dirty brown to a purplish hue; these cysts were of different sizes, some being as large as a pea, and others double or treble that size. A section made completely through the tumor, from without, revealed the following structures: Next to the skin a thick layer of fat; then a hard, flattened, fibrous mass; then the dense nodulated bodies above alluded to; and lastly, a layer of tissuc, somewhat hard in its character, and apparently fibrous, containing the cysts.

The layer of hardened tissue immediately subjacent to the superficial fascia, proved, when examined by the microscope, to be fibrous; the fibres interlacing closely one with another. By the addition of acetic acid the whole mass was rendered transparent, and scattered throughout might be seen numerous elongated oval nuclei, about the 1-1500th part of an inch in length, and about the 1-4000th part of an inch in breadth. (See fig. 4, *a.*)

Fig. 4.

*a.* Fibrous structure, showing oval nuclei from tumour of the breast, after the addition of acetic acid; magnified 220 diameters.

*b.* Fat vesicles, showing crystals of margaric acid.

*c.* Oil globules, and granular matter.

The nodulated masses presented the same fibrous appearance; no nuclei, however, could be detected even after the addition of acetic acid. The mass in which the cysts occurred, and which was situated directly above the great pectoral muscle, proved, upon examination, to be composed of merely condensed, adipose tissue; the fat vesicles being flattened, and arranged in laminæ, one upon another; giving the whole a somewhat fibrous appearance. Many of the fat vesicles contained crystalline, stellated nuclei, of margaric acid. (See fig. 4, *b.*) I use the term nuclei, although strictly speaking, it is hardly a proper expression, inasmuch as these crystals are deposited subsequently to the formation of the vesicle, and in reality have nothing whatever to do with the production of the walls of the cell. In some instances, the crystals seemed to be deposited on the external surface of the cell wall. The cysts, which were filled with an oily fluid, were surrounded by fibrous walls, and I was not able to detect, after the most careful examination, any vestige of a secreting, lining, epithelial membrane. The contents of the cysts consisted chiefly of oil globules; but there was also a considerable quantity of granular matter. (Fig. 4, *c.*)

The substance of the mammary gland seemed to have been almost entirely absorbed; very few of the glandular cells could be detected, and those were situated only in the anterior portion of the breast; in this region the galactophorous tubes remained in a healthy condition.

An analogy has been pointed out by some of the late German pathologists, between cirrhosis of the liver, and that condition of the mammary gland which we have just seen, in which we have great and hypertrophic development of the fibrous sheath of the gland; this sheath covering not only the external surface, but also sending in throughout the whole gland, prolongations of thick membraneous fascia, permeating its substance in the same manner as the capsule of Glisson does that of the liver. The same causes acting upon analogous tissues, will produce like results; the development of a fibrous tumor causing atrophy and absorption, more or less complete, of either gland.

The occurrence of the peculiar oily cysts is a circumstance admitting of by no means an easy explanation. It is no uncommon thing to meet with cysts full of oily contents, formed by the dilatation of the galactophorous tubes; but this was not the case in this instance: here the cysts were situated at the deepest seated portion of the tumor, and were entirely insulated, and appeared to be destitute of any epithelial lining membrane. Is it not possible for these cysts to have been produced mechanically, by the pressure of a superincumbent fibrous mass, upon a layer of adipose tissue, resting against the anterior wall of the thorax, so causing the rupture of the fat vesicles, and allowing the free oil globules to escape? We know that such will be the case, if the fat vesicles be compressed under the field of the microscope, between two plates of glass; and it can easily be conceived possible, that sufficient pressure should be produced by the tension of the integuments, transmitted downwards, through a hard, resisting, fibrous tumor. And if we add to this, the expansive force of a rapidly developing fibrous growth, it seems to me that we have causes quite sufficient to produce the results which in this case we have seen were effected.

## Figures and Tables

**Fig. 3. f1:**
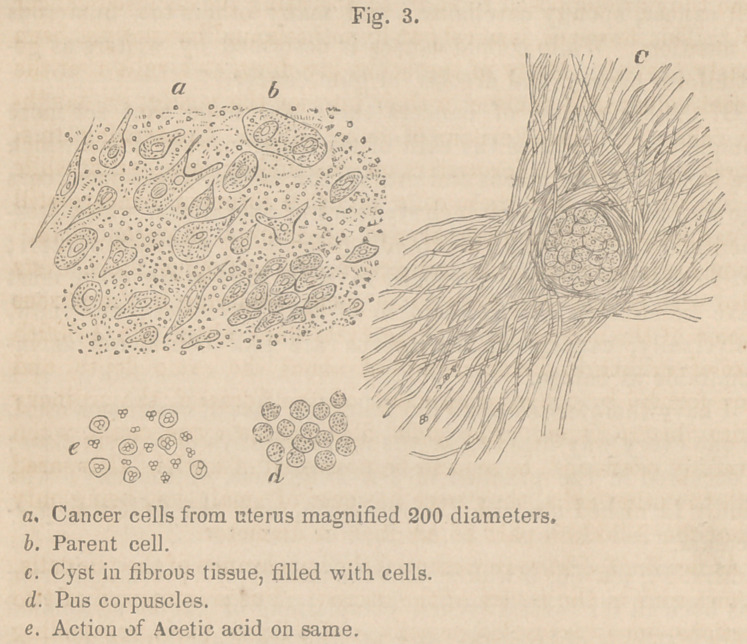


**Fig. 4. f2:**